# Supplementary material for: Content-rich biological network constructed by mining PubMed abstracts
Source: BMC Bioinformatics. 2004 Oct 8;5:147. doi: 10.1186/1471-2105-5-147 (PMC528731; doi:10.1186/1471-2105-5-147)
Supplement: Additional File 5 — The original Chilibot query results of the term "long-term potentiation (LTP)" and 22 other terms, limiting the latest references analyzed to the years 1990, 1995, 2000, and 2004. [file 1471-2105-5-147-S5.bz2 › chilibotAdditionalFile5/ltp2000/html/CREB_PI-3K.html]

 


 **CREB** and **PI-3K** 
  
Found 8 abstracts in PubMed,  **8 abstracts were retrieved and analyzed**.  


---

 Search Google  |
 PDF files only 
|  EDU domain only 

---

**Interactive relationship** (e.g. stimulation, inhibition, etc)

- However, AMPA receptor stimulation led to a calcium dependent phosphorylation of the nuclear transcription factor  **CREB** , which could be prevented by inhibitors of MEK or PI 3 kinase  [ **PI-3K** ] .  Ref: 10407026 J Neurosci, 1999
- Further, PDGF, PI 3 kinase  [ **PI-3K** ] , and Rac1 each activated the cyclin D 1 promoter at the cyclic adenosine monophosphate response element binding protein  **CREB**  activating transcription factor ATF 2 binding site, as evidenced by expression of a  **CREB**  ATF 2 reporter plasmid.  Ref: 11017907 Am J Respir Cell Mol Biol, 2000
- Constitutively active and dominant negative regulators of the Ras and PI 3 kinase  [ **PI-3K** ]  pathways confirmed the contribution of these pathways for  **CREB**  regulation by IGF I.  Ref: 9915817 J Biol Chem, 1999

**Parallel relationship** (e.g. studied together, co-existance, homology, etc.)

- Finally, PI 3 kinase  [ **PI-3K** ]  and Rac1 induced  **CREB**  ATF 2 transactivation were each inhibited by catalase.  Ref: 11017907 Am J Respir Cell Mol Biol, 2000
- Inhibition of MAPK kinase with PD98059, PI 3 kinase  [ **PI-3K** ]  with wortmannin, and p38 MAPK with SB203580 blocked IGF I mediated phosphorylation and transcriptional activation of  **CREB**  by 30 50% p <.  Ref: 9915817 J Biol Chem, 1999
